# Chorioretinal thinning in chronic kidney disease links to inflammation and endothelial dysfunction

**DOI:** 10.1172/jci.insight.89173

**Published:** 2016-12-08

**Authors:** Craig Balmforth, Job J.M.H. van Bragt, Titia Ruijs, James R. Cameron, Robert Kimmitt, Rebecca Moorhouse, Alicja Czopek, May Khei Hu, Peter J. Gallacher, James W. Dear, Shyamanga Borooah, Iain M. MacIntyre, Tom M.C. Pearson, Laura Willox, Dinesh Talwar, Muriel Tafflet, Christophe Roubeix, Florian Sennlaub, Siddharthan Chandran, Baljean Dhillon, David J. Webb, Neeraj Dhaun

**Affiliations:** 1 BHF Centre of Research Excellence, University of Edinburgh, The Queen’s Medical Research Institute, Edinburgh,; 2 Anne Rowling Regenerative Neurology Clinic, Centre for Clinical Brain Sciences, University of Edinburgh,; 3 Princess Alexandra Eye Pavilion, Edinburgh, United Kingdom.; 4 Department of Clinical Biochemistry and Metabolic Medicine, Royal Infirmary of Glasgow, United Kingdom.; 5 INSERM Unit 970, Paris Cardiovascular Research Center – PARCC and Descartes University, Paris, France.; 6 Institut National de la Santé et de la Recherche Médicale, Institut de la Vision, Paris, France.

## Abstract

**BACKGROUND.** Chronic kidney disease (CKD) is strongly associated with cardiovascular disease and there is an established association between vasculopathy affecting the kidney and eye. Optical coherence tomography (OCT) is a novel, rapid method for high-definition imaging of the retina and choroid. Its use in patients at high cardiovascular disease risk remains unexplored.

**METHODS.** We used the new SPECTRALIS OCT machine to examine retinal and retinal nerve fiber layer (RNFL) thickness, macular volume, and choroidal thickness in a prospective cross-sectional study in 150 subjects: 50 patients with hypertension (defined as a documented clinic BP greater than or equal to 140/90 mmHg (prior to starting any treatment) with no underlying cause identified); 50 with CKD (estimated glomerular filtration rate (eGFR) 8–125 ml/min/1.73 m^2^); and 50 matched healthy controls. We excluded those with diabetes. The same, masked ophthalmologist carried out each study. Plasma IL-6, TNF-α , asymmetric dimethylarginine (ADMA), and endothelin-1 (ET-1), as measures of inflammation and endothelial function, were also assessed.

**RESULTS.** Retinal thickness, macular volume, and choroidal thickness were all reduced in CKD compared with hypertensive and healthy subjects (for retinal thickness and macular volume *P* < 0.0001 for CKD vs. healthy and for CKD vs. hypertensive subjects; for choroidal thickness *P* < 0.001 for CKD vs. healthy and for CKD vs. hypertensive subjects). RNFL thickness did not differ between groups. Interestingly, a thinner choroid was associated with a lower eGFR (*r* = 0.35, *P* <0.0001) and, in CKD, with proteinuria (*r* = –0.58, *P* < 0.001) as well as increased circulating C-reactive protein (*r* = –0.57, *P* = 0.0002), IL-6 (*r* = –0.40, *P* < 0.01), ADMA (*r* = –0.37, *P* = 0.02), and ET-1 (*r* = –0.44, *P* < 0.01). Finally, choroidal thinning was associated with renal histological inflammation and arterial stiffness. In a model of hypertension, choroidal thinning was seen only in the presence of renal injury.

**CONCLUSIONS.** Chorioretinal thinning in CKD is associated with lower eGFR and greater proteinuria, but not BP. Larger studies, in more targeted groups of patients, are now needed to clarify whether these eye changes reflect the natural history of CKD. Similarly, the associations with arterial stiffness, inflammation, and endothelial dysfunction warrant further examination.

**TRIAL REGISTRATION.** Registration number at www.clinicalTrials.gov: NCT02132741.

**SOURCE OF FUNDING. **TR was supported by a bursary from the Erasmus Medical Centre, Rotterdam. JJMHvB was supported by a bursary from the Utrecht University. JRC is supported by a Rowling Scholarship. SB was supported by a Wellcome Trust funded clinical research fellowship from the Scottish Translational Medicine and Therapeutics Initiative, and by a Rowling Scholarship, at the time of this work. ND is supported by a British Heart Foundation Intermediate Clinical Research Fellowship (FS/13/30/29994).

## Abstract 2

Chorioretinal thinning relates to the degree of inflammation and kidney injury in patients with kidney disease.

## Introduction

Cardiovascular disease (CVD) remains the leading cause of death worldwide, contributing to ~30% of all deaths globally in 2010 (1). Arterial hypertension is a major risk factor for atherosclerosis, coronary artery disease, stroke, and chronic kidney disease (CKD), and is one of the most prominent contributors to CVD death worldwide (2). Currently, it is estimated that a quarter of the world’s adult population is hypertensive and this number is projected to rise to nearly 30% by 2025 (3). CKD is also common, affecting 6%–11% of the world’s population (4), and is strongly associated with incident CVD (5). Hypertension is an independent risk factor for CKD progression (6), and is a frequent finding in patients with CKD (7).

Renal microvascular changes are considered important in the development of CKD. Currently, these can only be assessed reliably through renal biopsy, which is not without risk. Furthermore, interval renal biopsy, to assess changes in the kidney’s microvasculature over time and in response to therapy, is impractical. The kidney and eye are remarkably similar in their developmental, structural, and pathogenic pathways (8). Interestingly, the renal podocyte is very similar in structure (possessing a large surface area with interdigitating foot processes) and function to the vascular pericyte (8), so diseases may manifest themselves similarly. Transparency of the ocular media offers a unique opportunity to directly visualize and image chorioretinal microvasculature structures within the eye that may be affected in systemic diseases such as arterial hypertension (9) and CKD (10).

Optical coherence tomography (OCT) is a novel, noninvasive, and rapid method for cross-sectionally imaging the retina and choroid (11). Recent advances in OCT technology have led to the introduction of spectral domain OCT (SD-OCT). In combination with an enhanced depth imaging (EDI) feature, SD-EDI-OCT enables the identification of specific cell layers within the retina in high resolution, as well as deeper structures such as the choroid, in a way only previously possible in histological samples. The utility of SD-EDI-OCT has not previously been reported in patients with hypertension or those with hypertension and CKD. We hypothesized that OCT imaging would report structural changes within the retina and choroid in patients with hypertension and CKD, in keeping with an increased risk of CVD, compared with matched, healthy controls. Based on our observations, we further explored the mechanistic roles of inflammation and endothelial dysfunction in these changes.

## Results

Fifty patients with hypertension, 50 patients with varying degrees of CKD, and 50 healthy controls were recruited into the studies. Subject demographics and medications are shown in Table 1. CKD patients’ diagnoses are shown in Supplemental Table 1; supplemental material available online with this article; doi:10.1172/jci.insight.89173DS1.

**Figure 1:**
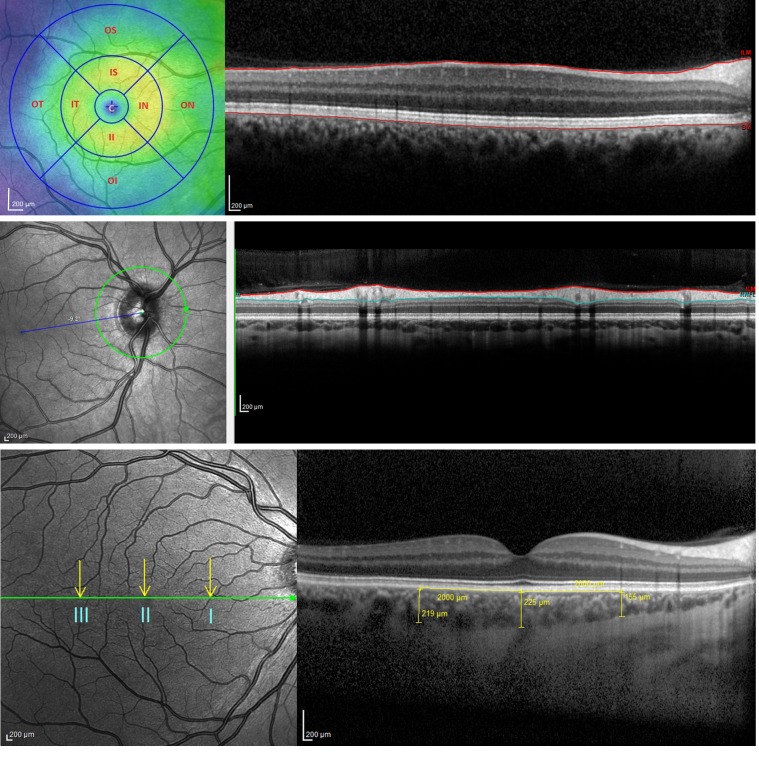
This figure shows the chorioretinal structures *en face* (left images) and as a cross section (right images). The Early Treatment Diabetic Retinopathy Study map divides the macula into 9 subfields. A circular grid is centered over the fovea and consists of 3 concentric rings of diameters 1, 3, and 6 mm, respectively. (**A**) The inner and outer rings are further divided into quadrants: temporal, nasal, superior, and inferior (left; see also Figure 2). Retinal thickness was defined as the area between the internal limiting membrane (ILM) and the hyporeflective line between the retinal pigment epithelium (RPE) and the choriocapillaries (right). (**B**) Retinal nerve fiber layer thickness was defined as the area bordered in red. (**C**) Choroidal thickness was measured at 3 locations on the macula: I = 2 mm nasal to the fovea, II = subfoveal, III = 2 mm temporal to the fovea. Scale bars: 200 μm.

**Figure 2:**
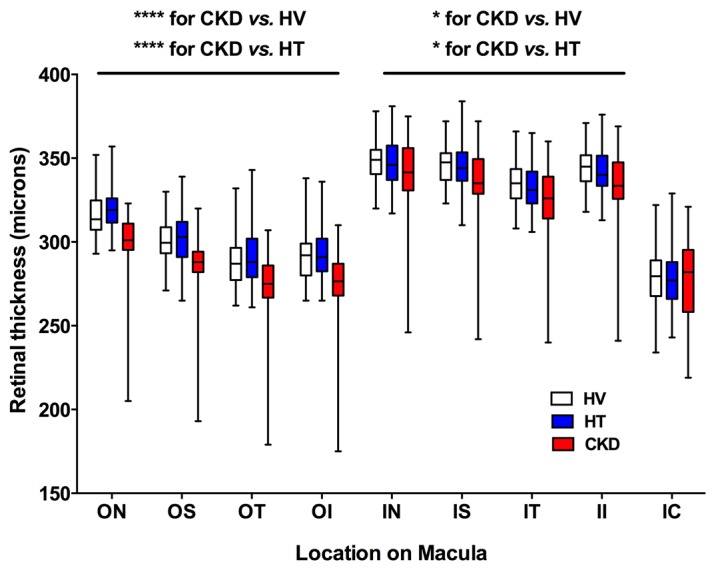
Retinal thickness as a function of macular location. Box-and-whisker plots showing retinal thickness in subjects with hypertension (HT), chronic kidney disease (CKD), and healthy volunteers (HV) across different areas of the macula (see Figure 1A): ON, outer nasal; OS, outer superior; OT, outer temporal; OI, outer inferior; IN, inner nasal; IS, inner superior; IT, inner temporal; II, inner inferior; IC, inner circle. At each of the 4 locations ON, OS, OT, and OI, ^****^P < 0.0001 for CKD vs. healthy and for CKD vs. hypertensive subjects. At each of the 5 locations IN, IS, IT, II, IC, ^*^P < 0.05 for CKD vs. healthy and for CKD vs. hypertensive subjects. The box-and-whisker plots display the first and third quartiles, with the line within the box representing the median value. The whiskers denote the minimum and maximum values. Thicknesses were examined by 2-way ANOVA, comparing thicknesses between all 3 patient groups at each macular location using Tukey correction for multiple comparisons. *n* = 50 subjects per group.

**Figure 3:**
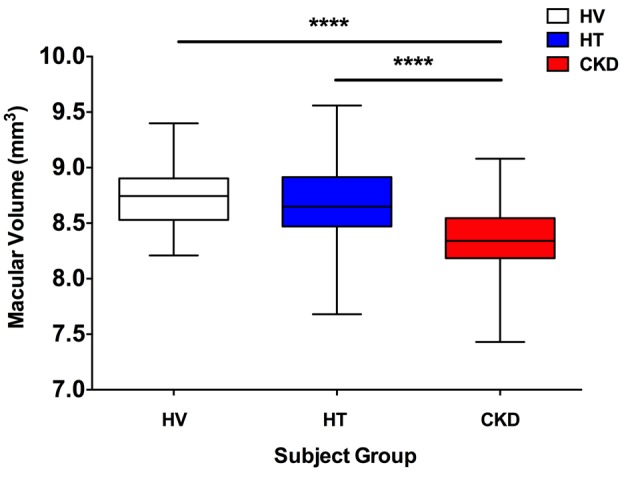
Macular volume. Box-and-whisker plots showing macular volume in subjects with hypertension (HT), chronic kidney disease (CKD), and healthy volunteers (HV). ^****^P < 0.0001 for CKD vs. healthy and for CKD vs. hypertensive subjects. The box-and-whisker plots display the first and third quartiles, with the line within the box representing the median value. The whiskers denote the minimum and maximum values. Volumes were examined by 1-way ANOVA, comparing thicknesses between all 3 patient groups at each macular location using Tukey correction for multiple comparisons. *n* = 50 subjects per group.

Figure 1 shows the chorioretinal structures and measurements of interest. Retinal thickness did not differ between healthy volunteers and subjects with hypertension. However, patients with CKD had a thinner retina at both inner and outer locations of the Early Treatment Diabetic Retinopathy Study (ETDRS) map on the macula; retinal thinning was particularly apparent at the 4 outer locations — outer nasal, outer superior, outer temporal, and outer inferior (Figure 2, P** < 0.0001 for CKD vs. healthy and for CKD vs. hypertensive subjects). The retina was ~10% thinner at these outer positions in CKD compared with the other 2 groups, and at all positions women had thinner retinas than men (*P* < 0.05). In keeping with a thinner retina, CKD patients had a reduced macular volume compared with both patients with hypertension and healthy volunteers (*P* < 0.0001 for both) (Figure 3). However, macular volume did not differ between healthy and hypertensive subjects. In both healthy and hypertensive subjects, age correlated with retinal thickness (at all locations) and with macular volume, such that older subjects had thinner retinas and lower macular volume (data not shown). In CKD, retinal thickness was not associated with age. However, macular volume was reduced in older subjects (*r* = –0.33, *P* = 0.02). Neither retinal thickness nor macular volume related to systolic BP (SBP), diastolic BP (DBP), estimated glomerular filtration rate (eGFR), or proteinuria in any of the 3 subject groups. For macular volume, the area under the receiver operator curve (ROC) was 0.82 (95% CI 0.74–0.91; curve not shown). Supplemental Table 2 lists the derived sensitivities and specificities at different cutoff values for macular volume. A value of less than 8.55 mm^3^ yielded good sensitivity and specificity for the detection of CKD.

**Figure 4:**
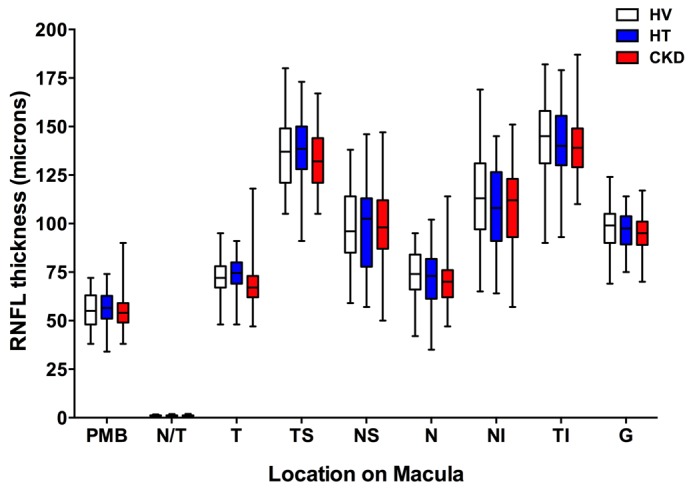
Retinal nerve fiber thickness vs. **macular location.** Box-and-whisker plots showing retinal nerve fiber layer (RNFL) thickness in subjects with hypertension (HT), chronic kidney disease (CKD), and healthy volunteers (HV) across different areas of the macula: T, temporal; TS, temporal-superior; NS, nasal-superior; N, nasal; NI, nasal-inferior; TI, temporal-inferior. PMB, papillo-macular bundle; N/T, nasal-temporal ratio; G, average RNFL thickness. The box-and-whisker plots display the first and third quartiles, with the line within the box representing the median value. The whiskers denote the minimum and maximum values. Thicknesses were examined by 2-way ANOVA, comparing thicknesses between all 3 patient groups at each macular location using Tukey correction for multiple comparisons. *n* = 50 subjects per group.

**Figure 5:**
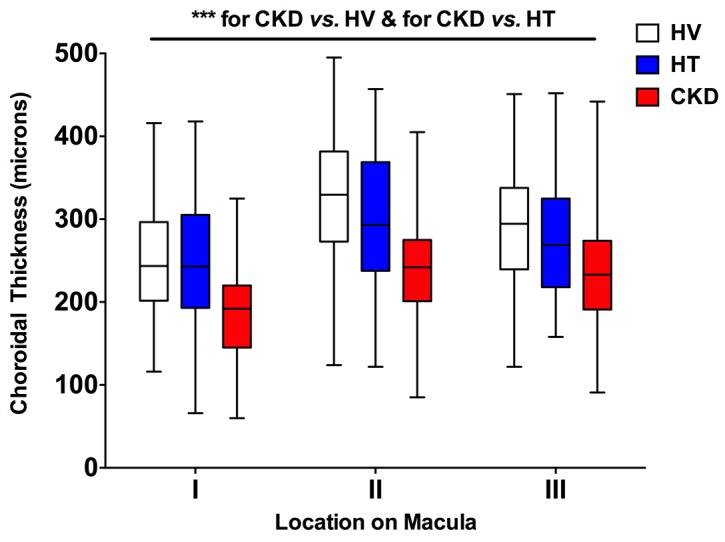
Choroidal thickness vs. macular location. Box-and-whisker plots showing choroidal thickness in subjects with hypertension (HT), chronic kidney disease (CKD), and healthy volunteers (HV) across 3 locations on the macula: I = 2 mm nasal to the fovea, II = subfoveal, III = 2 mm temporal to the fovea. At each of these 3 locations, ^***^P < 0.001 for CKD vs. healthy and for CKD vs. hypertensive subjects. The box-and-whisker plots display the first and third quartiles, with the line within the box representing the median value. The whiskers denote the minimum and maximum values. Thicknesses were examined by 2-way ANOVA, comparing thicknesses between all 3 patient groups at each macular location using Tukey correction for multiple comparisons. *n* = 50 subjects per group.

Retinal nerve fiber layer (RNFL) thickness did not differ between the 3 subject groups studied (Figure 4). Choroidal thickness did not differ between healthy volunteers and patients with hypertension at the 3 locations assessed (Figure 5; location I was 2 mm nasal to the fovea; location II was directly over the fovea (subfoveal choroidal thickness); location III was 2 mm temporal to the fovea). Interestingly, CKD was associated with a thinner choroid at each of these locations compared with healthy and hypertensive patients (*P* < 0.001 for both), a reduction in choroidal thickness of ~15%–20% (Figure 5 and Supplemental Videos 1–4). For subfoveal choroidal thickness, the area under the ROC was 0.81 (95% CI 0.72–0.89; curve not shown). Supplemental Table 2 lists the derived sensitivities and specificities at different cutoff values for choroidal thickness. A value of less than 277 μm yielded good sensitivity and specificity for the detection of CKD.

### Choroidal thickness in CKD.

In patients with hypertension and CKD, increasing age was associated with a thinner choroid (*r* = –0.55, *P* < 0.001 for locations I, II, and III for hypertension; *r* = –0.38, *P* < 0.05 for locations I, II and III for CKD) but this was not the case in healthy subjects. However, SBP correlated inversely with choroidal thickness in healthy (*r* = –0.32, *P* < 0.05 for locations II and III) but not in diseased subjects; DBP did not correlate with choroidal thickness in any of the 3 groups.

**Figure 6:**
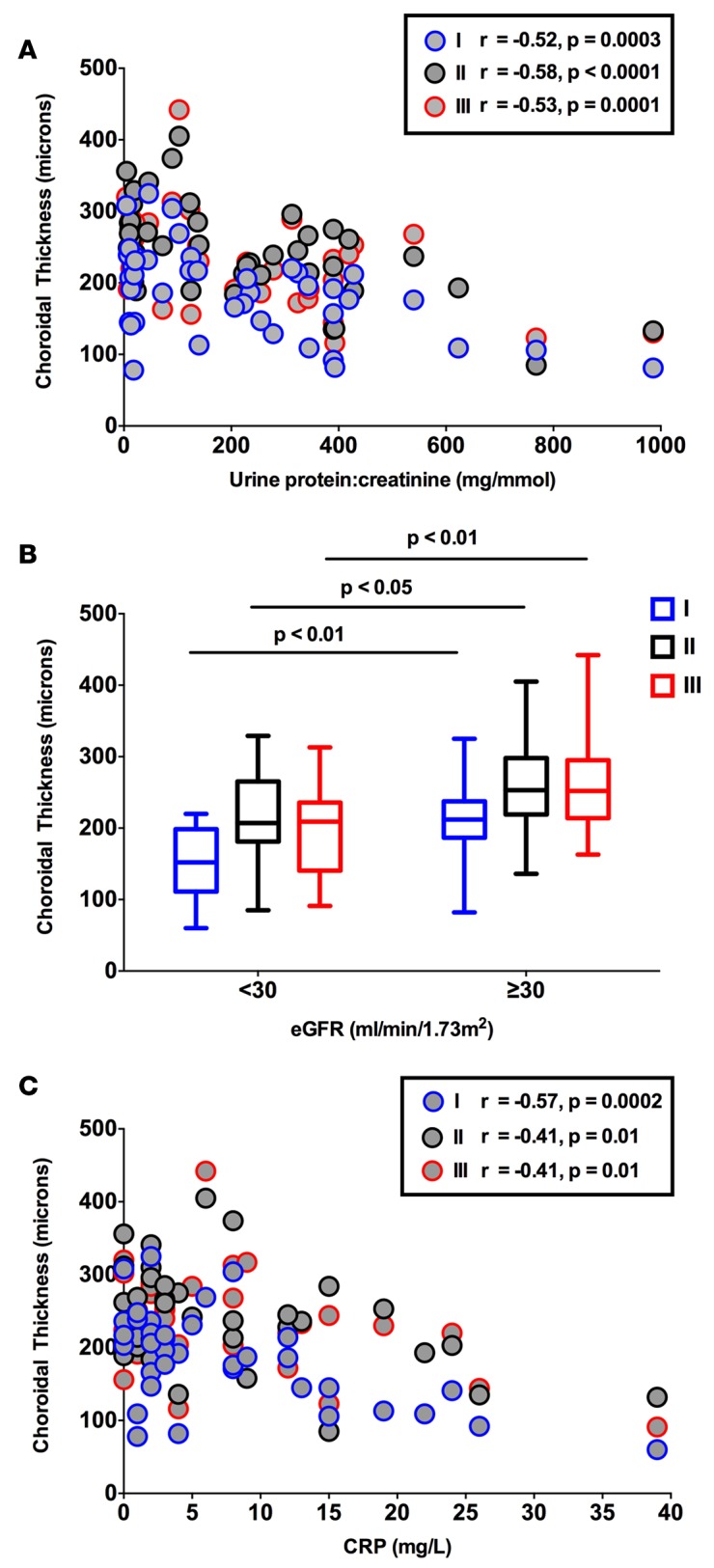
Correlations of choroidal thickness with clinical measures. Correlation of choroidal thickness (at locations I, II, and III on the macula), with (**A**) C-reactive protein (CRP) (Pearson’s correlation), (**B**) estimated glomerular filtration rate (eGFR), and (**C**) proteinuria (Spearman’s correlation) in patients with chronic kidney disease. eGFR was calculated using the Modification of Diet in Renal Disease (MDRD) equation. Proteinuria was quantified on the basis of a urine total protein/creatinine ratio (P:Cr). A P:Cr of greater than 15 mg/mmol is abnormal and greater than 300 is considered within the nephrotic range. The box-and-whisker plots in **B** display the first and third quartiles, with the line within the box representing the median value. The whiskers denote the minimum and maximum values. *n* = 50 subjects per group. Statistically significant differences were assessed with a 2-way ANOVA with a Turkey correction for multiple comparisons

In those with CKD, a thinner choroid — at each of the 3 locations assessed — was also associated with a higher serum C-reactive protein (CRP) concentration, a lower eGFR, and greater degrees of proteinuria (Figure 6). Interestingly, when all subjects were included in the analysis, this correlation with eGFR remained (Supplemental Figure 1). Based on these observations, we measured plasma IL-6 and TNF-α, both important mediators of systemic inflammation (12), in all subjects. As eGFR and proteinuria are strong, independent vascular risk factors (13), we also went on to assess plasma endothelin-1 (ET-1) and asymmetric dimethylarginine (ADMA) as measures of endothelial function.

**Figure 7:**
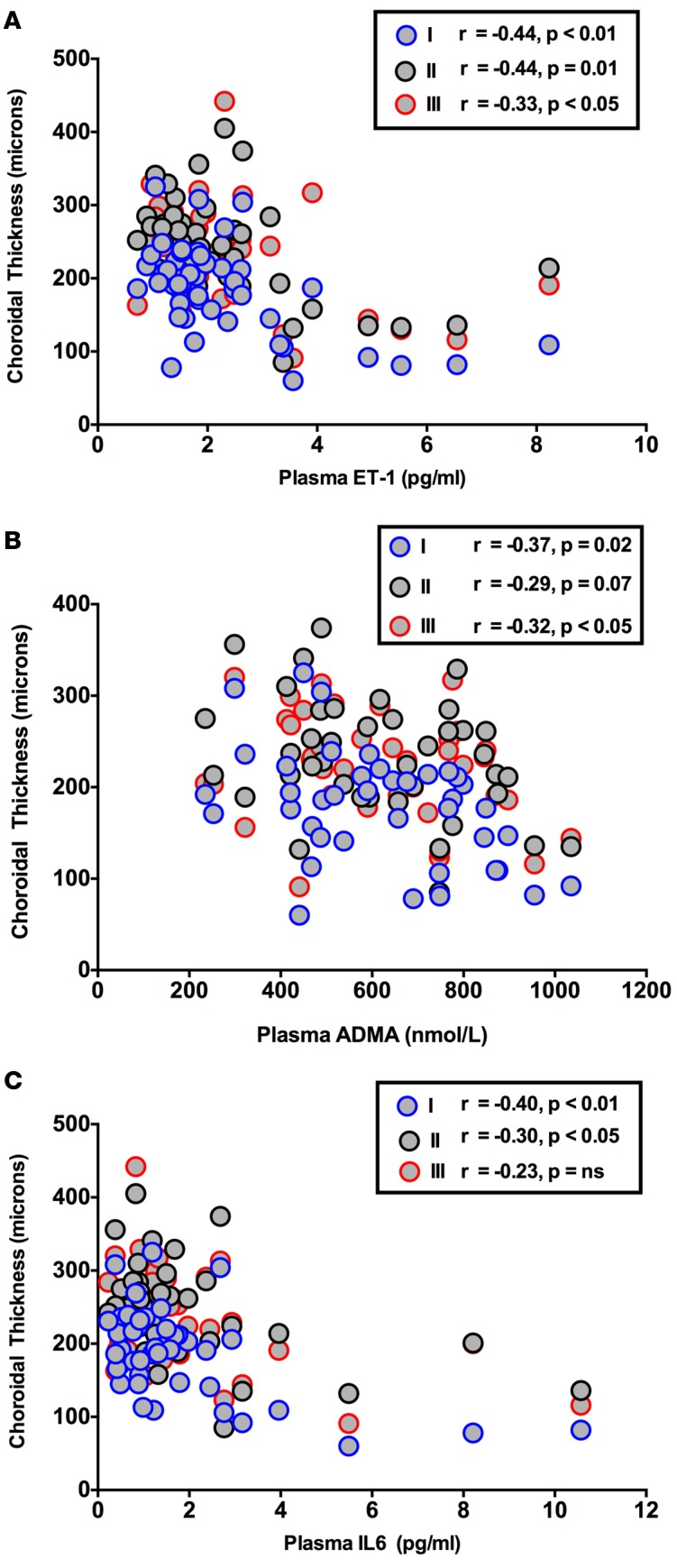
Correlations of choroidal thickness with nontraditional cardiovascular risk factors. Correlation of choroidal thickness (at locations I, II, and III on the macula), with (**A**) IL-6, (**B**) endothelin-1 (ET-1), and (**C**) asymmetric dimethylarginine (ADMA) in patients with chronic kidney disease. Correlation coefficients are Spearman’s coefficients. *n* = 50 subjects per group.

Plasma IL-6 was higher in those with hypertension and CKD compared with healthy subjects (Table 2, *P* < 0.05) but did not differ between hypertension and CKD. Whereas IL-6 did not associate with choroidal thickness in healthy and hypertensive subjects, a thinner choroid, at all 3 locations, correlated with a higher concentration of IL-6 in CKD (Figure 7A). Circulating TNF-α was higher in CKD compared with both healthy and hypertensive subjects Table 2, *P* < 0.0001 for both), but unlike IL-6 did not associate with choroidal thickness in those with CKD (or in healthy and hypertensive subjects).

Plasma ET-1 was higher in CKD than in healthy and hypertensive patients (Table 2, *P* < 0.05 for both) and also correlated with choroidal thickness in these patients (Figure 7B), such that a higher circulating ET-1 was associated with a thinner choroid. Finally, plasma ADMA, which was also higher in those with CKD compared with both healthy and hypertensive subjects (Table 2, *P* < 0.0001 for both), also correlated inversely with choroidal thickness (Figure 7C). ET-1 and ADMA showed no associations with choroidal thickness in healthy and hypertensive subjects.

Including only those variables with a linear relationship with choroidal thickness into multivariable analysis (age, CRP, urine protein/creatinine ratio [P:Cr], IL-6, ET-1, and ADMA) demonstrated that CRP, proteinuria, and IL-6 were independent predictors of choroidal thickness in CKD at locations I and II. Proteinuria was also an independent predictor in location III, as was age (Table 3).

Given the intercorrelation of the variables included in multivariable analysis we went on to utilize a principal component analysis strategy (Supplemental Figure 2). In order to enter normally distributed variables into the analysis we first log transformed eGFR, proteinuria, CRP, IL-6, and ET-1. The first 3-component axis explained 68.4% of the total variance, and the first 2-component axis 57.5%. The analysis showed a slight opposition in the choroidal location with ADMA, ET-1, IL-6, and proteinuria (first principal component). The analysis did not show a different opposition in the 3 choroidal locations and these variables. This implies that no one choroidal location was more associated than any other with any of the variables assessed. Furthermore, the analysis showed that none of the variables were redundant (except at locations II and III, which are very close in the first and second axis).

### Chorioretinal thinning, renal inflammation, and arterial stiffness.

Given the associations between chorioretinal thicknesses and measures of systemic inflammation, we explored these relationships with renal histological inflammation. In patients with antineutrophil cytoplasmic autoantibody (ANCA) vasculitis, an autoimmune disorder characterized by intense small vessel inflammation in multiple organs often including the kidneys, the extent of glomerular histological inflammation — focal necrotizing lesions and cellular crescents (Supplemental Figure 3, A and B) — did not correlate with retinal thickness. Interestingly, we did find a correlation with choroidal thickness, such that a greater degree of glomerular inflammatory injury was associated with a thinner choroid (Supplemental Figure 3, C and D).

Finally, 20 of our 50 CKD subjects had had previous assessment of arterial stiffness (14, 15) using the gold-standard measure of pulse wave velocity (PWV) (16). Increased arterial stiffness is a recognized marker of CVD risk (17). The endothelium is an important regulator of arterial stiffness (18). Increasing vascular stiffness as reflected by a higher PWV was associated inversely with choroidal thickness (Supplemental Figure 4).

### Choroidal thinning in mice with hypertension and renal injury.

To explore whether we might be able to perform future mechanistic studies to better understand our clinical data, we aimed to use OCT to measure chorioretinal thicknesses in mice with either hypertension alone or hypertension with associated renal injury. Mice with hypertension alone showed no chorioretinal thinning. By comparison, and in keeping with our clinical data, animals with matched hypertension and renal injury developed choroidal thinning (Supplemental Figure 5), although without evidence of retinal thinning.

## Discussion

We have shown, to our knowledge for the first time, that patients with varying degrees of predialysis CKD exhibit substantial retinal and choroidal thinning, alongside a reduction in macular volume, compared with age- and sex-matched healthy volunteers as well as matched subjects with hypertension. Interestingly, for the choroid, there were strong correlations between its thickness and the level of systemic inflammation (measured using high-sensitivity CRP [hsCRP]) and degree of renal dysfunction (represented by eGFR and proteinuria). Together, these findings suggest that the retinal and choroidal changes seen in those with CKD may be representative of a generalized systemic microvascular injury, and may also reflect underlying renal injury.

The Chronic Renal Insufficiency Cohort (CRIC) study, which assessed the relationship between CKD and retinal pathology, revealed a high prevalence of fundus pathology in CKD patients, probably because half of the patients had long-standing diabetes (10). Few studies have made OCT assessments in those with CKD and all have included only those with end-stage renal failure (ESRF — mostly due to diabetes) requiring renal replacement therapy (19–21). Thus, ours is possibly the first study to investigate OCT measures in those with predialysis CKD. The strong correlations for the associations seen, despite the group of CKD patients not comprising a single disease etiology, suggests that these may be a general feature of predialysis CKD and relatively independent of underlying diagnosis. While previous studies examining the impact of dialysis on retinal and choroidal parameters have shown differences (20, 21), these may largely be attributable to the fluid shifts characteristic of dialysis. In another study, RNFL thickness was found to be reduced in dialysis patients (with ESRF due to reasons other than diabetes) compared with controls (19). In our own study we found no difference in RNFL thickness in CKD compared with both healthy and hypertensive subjects, but it may be that optic neuropathy is a feature of late CKD. In keeping with other work, our data confirm a negative correlation between choroidal thickness and age (22).

Importantly, and in comparison with CKD, we found no differences between healthy and hypertensive subjects in any of the parameters studied. As for CKD, there are few studies that have examined OCT measures in patients with hypertension and only 1 with robust methodology (23). Here, Muraoka et al. showed that retinal arteriolar and venular wall thicknesses were increased in hypertensive compared with healthy subjects. The authors did not study the parameters assessed in the current study. Our own lack of differences may be due to a reasonably young cohort (mean age ~50 years) of solely patients of European descent who had no overt evidence of end-organ damage — although we only assessed kidney damage by means of serum creatinine (and eGFR) and urinalysis and there was no assessment of cardiac or cerebral function. These should be considered in future studies.

The retina is a complex neurovascular tissue. Photoreceptors resting on the retinal pigment epithelium form the outer layer, and the RNFL the innermost layer. Between the photoreceptors and RNFL are the neural processing cells including horizontal, amacrine, and bipolar cells synapsing with retinal ganglion cells whose axons form the RNFL. It is possible that the reduction in retinal thickness and macular volume seen in those with CKD is due to the atrophy of these cells probably as a result of compromised blood supply due to microvascular injury. The choroid is a largely vascular organ and so the thinning here is likely to reflect purely microvascular damage and loss. As seen in Figure 2, the reduction in retinal thickness in CKD patients was largely restricted to the outer retinal layers. Of note, the outer third of the retina receives its blood supply from the choroid (with the inner two-thirds being retinal in origin). Thus, it is possible the retinal changes occur secondary to choroidal pathology, compromising choroidal blood flow to the retina. This might also explain, in part, the lack of association between the extent of retinal thinning and measures such as CRP, eGFR, or proteinuria.

There are a number of potential explanations for the changes we have observed. Hypertension is an important and common contributor to microvascular injury in a number of organs including the kidney and the eye (2, 24). However, the retinal and choroidal thinning seen in CKD is unlikely to be related to BP because differences were not seen between the hypertensive cohort and healthy volunteers, even though all components of BP were significantly higher in the hypertensive group compared with those in our group with CKD. Furthermore, neither retinal nor choroidal thickness correlated with SBP or DBP in those with CKD. Dysfunction of the autonomic nervous system may contribute to the changes observed. CKD is a state of heightened sympathetic activity and this may contribute to disease progression (25). While the choroidal circulation has autonomic innervation, the retinal circulation does not. Thus, the thinning of the outer retina and choroid would be consistent with increased sympathetic tone affecting the choroidal vasculature. We did not investigate measures of sympathetic activity in the current study, but these would be an interesting area for future research.

However, we did see associations between systemic (and renal) inflammation and 2 important vascular risk factors, eGFR and proteinuria, and these may go some way in providing a mechanism to explain our observations. Inflammation is an important contributor to the development of cardiovascular and renal disease. hsCRP correlated inversely with choroidal thickness in CKD and was an independent predictor of thickness. On this basis, we assessed circulating IL-6 and TNF-α, both important mediators of inflammation in CVD and CKD. These were raised in CKD patients and IL-6 correlated inversely with choroidal thickness, consistent with its involvement in the process. Indeed, IL-6 is considered to be important in the pathogenesis of a number of eye conditions (26, 27) and is predictive of outcome in both CVD (28) and CKD (29). Therapeutic strategies blocking its effects are currently being explored in both eye (30) and cardiovascular (31) disorders, the pathology of which, based on the current data, may be linked.

Given the relationships seen between choroidal thickness and both eGFR and proteinuria we assessed endothelial function. Plasma ADMA, an endogenous inhibitor of nitric oxide synthase, and plasma ET-1 were measured as components of the nitric oxide and ET systems, respectively. Both contribute to vascular dysfunction in CKD and an imbalance (more ET-1/less nitric oxide) may contribute to vasoconstriction, inflammation, and atherosclerosis (32, 33). Circulating ADMA was ~2-fold higher in CKD than in both healthy and hypertensive subjects and, in keeping with previous studies, plasma ET-1 was also increased (34). Both ET-1 and ADMA associated strongly, and inversely, with choroidal thickness in those with CKD. ET-1 contributes to the vasoconstriction that is seen in many eye diseases (35, 36) and blocking its effects improves retinal vascular integrity (37). Thus, ET receptor antagonism, a novel therapeutic strategy currently being investigated for renoprotection in CKD (38), may also have benefits for the eye. Furthermore, given that there is often reciprocal upregulation of the nitric oxide system when the ET system is downregulated (32), an ET-blocking strategy may offset some of the potentially deleterious effects of elevated circulating ADMA.

As limitations, medications taken by our patients, such as angiotensin-converting enzyme inhibitors, β-blockers, and statins may have had effects on the OCT parameters studied. However, all patients were stabilized on their therapies and this is an unavoidable limitation of such studies. Furthermore, we observed undulation of the choroidal-scleral interface, limiting the power of single-point choroidal thickness measurements, and suggesting future studies should examine choroidal volume. This quantifies the overall disease burden and may be helpful in understanding disease pathophysiology and for assessing the response to treatment in chorioretinal disorders (39). Finally, our study describes associations and so these findings should be explored further following pharmacological interventions and renal transplantation. Our preclinical data are potentially the first to show that choroidal thickness can be assessed in mice and this lends itself to future mechanistic studies.

### Conclusions.

A growing number of studies support the link between diseases affecting the eye and the kidney. As is the case for CKD, common eye diseases such as age-related macular degeneration (AMD) and glaucoma are strongly associated with age and vascular risk factors such as hypertension, diabetes, and smoking (8). Furthermore, pathology within the eye, such as retinal microvascular changes, is predictive of incident CKD, and patients with CKD may be at higher risk for eye diseases such as AMD and glaucoma (8). It would be of major clinical value if retinal OCT-derived metrics could be used to detect and monitor vascular injury within the eye at any early stage and as a surrogate measure of renal vascular injury. A recent study has suggested that retinal photography may be useful alongside proteinuria quantification in risk-stratifying CKD patients in terms of disease progression (40). The current data highlight the need for larger studies in a similarly diverse CKD population using standardized OCT measurements as well as preclinical studies to explore the mechanisms responsible for the changes seen. Such studies would need to assess whether OCT measures could provide information on CKD progression beyond that already provided by proteinuria and eGFR. They should also establish whether OCT imaging — a quick, simple, and noninvasive procedure — might be of value clinically in assisting diagnosis of systemic microvascular disease, assessing response to therapy, and in the earlier identification of patients at increased risk of CVD.

## Methods

See Supplemental Information for more information.

### Subjects.

This was a prospective, cross-sectional controlled study performed at the Anne Rowling Regenerative Neurology Clinic at the Royal Infirmary of Edinburgh. Subjects were recruited from the hypertension and renal outpatient clinics at the Royal Infirmary of Edinburgh and Western General Hospital, Edinburgh. Age- and sex-matched controls without any comorbidity were recruited from the community. We used a frequency matching protocol for the recruitment of patients with hypertension and healthy volunteers. For every 5 patients with CKD recruited we matched these, based on age and sex, with 5 hypertensive patients and 5 healthy volunteers. Hypertension was defined as a documented clinic BP greater than or equal to 140/90 mmHg (prior to starting any treatment) with no underlying cause identified. Both incident and prevalent patients with hypertension were included. Renal patients were categorized into the 5 stages of CKD on the basis of the Kidney Disease Outcome Quality Initiative (K/DOQI) classification (7). We excluded those with any eye disease, previous eye surgery, refractive error greater than ± 6 diopters, those with diabetes mellitus (excluded both on the basis of the medical history and by checking a fasting glucose that had to be < 6 mmol/l), and clinically overt CVD.

### Study protocol.

All subjects abstained from alcohol-, nicotine-, and caffeine-containing products for 24 hours, and food for 4 hours, prior to the study but continued their normal medications (except diuretics, which were omitted on the study day). All studies were performed at the same time of day in a quiet, temperature-controlled room using the same OCT equipment by the same ophthalmologist who was masked to study group. SBP and DBP were recorded in duplicate, with an appropriately sized cuff, using a validated oscillometric sphygmomanometer, the Omron HEM-705CP (41), and values are presented as the average of 2 recordings. Creatinine clearance, as an estimate of GFR, was calculated according to the Chronic Kidney Disease Epidemiology Collaboration (CKD-EPI) equation (42).

All subjects underwent a single examination of both eyes using the Heidelberg SPECTRALIS Spectral-Domain OCT machine (software version 6.0, Heidelberg Engineering). Each examination comprised 3 scan protocols for each eye: (a) a horizontal line scan through the macula, centered over the fovea, with EDI enabled for greater choroidal visualization; (b) a macular volume scan consisting of 61 horizontal B-scans with a separation of 120 μm covering the whole macular area; and (c) a peripapillary circular line scan centered over the optic disc, with Nsite Axonal Analytics software automated segmentation of the RNFL (43).

Measurements recorded included retinal thickness, RNFL thickness, macular volume, and choroidal thickness. The images took advantage of the proprietary TruTrack active eye tracking and Automatic Real-Time (ART) software, which averages the image over 100 scans, to generate a single high-resolution scan image. OCT measurements of retinal thickness were performed according to the ETDRS protocol (44). The ETDRS map divides the macula into 9 subfields. The circular grid is centered over the fovea and consists of 3 concentric rings of diameters 1, 3, and 6 mm, respectively. The inner and outer rings are further divided into quadrants: temporal, nasal, superior, and inferior (Figure 1). The retinal thickness, RNFL thickness, and macular volume were measured using the automatic segmentation values of the SPECTRALIS OCT. The choroidal thickness was measured manually, on the horizontal EDI line scan, in 3 separate locations: subfoveal, and 2 mm nasal and 2 mm temporal to the fovea. The measurement was taken in a vertical line from the outer hyper-reflective line corresponding to the base of the retinal pigment epithelium (RPE) (RPE/basement membrane complex), to the choroidal-scleral junction (Figure 1).

### Sample collection and analysis.

Venous blood samples for ADMA, ET-1, IL-6, and TNF-α were collected in EDTA tubes. These were immediately centrifuged at 2,500 *g* for 20 minutes at 4°C. Samples were stored at –80°C until analysis.

Plasma ADMA concentrations were measured using an optimized and fully validated high-performance liquid chromatography method, as previously described (45) (intra- and interassay variations 1.9% and 2.3%, respectively). Plasma ET-1, TNF-α, and IL-6 were determined by ELISA (R&D Systems). The mean recovery of ET-1 was greater than 95%. The intra- and interassay variations were 4% and 6%, respectively. The cross-reactivity of the assay was 23% for ET-2, 0.5% for ET-3, and there was no cross-reactivity with big ET-1. For TNF-α, the mean recovery from plasma was 99%. The intra- and interassay variations were 6% and 8%, respectively. The cross-reactivity of the assay was less than 0.5% with related molecules. For IL-6 the mean recovery was 97% with intra- and interassay variations of 7%. The cross-reactivity of the assay was less than 0.5% with related molecules. Serum hsCRP concentrations were quantified in the hospital biochemistry laboratory using a validated latex particle–enhanced immunoturbidimetry technique (Vitros 5, 1 FS Chemistry Systems, Ortho-Clinical Diagnostics, Inc.) (intra- and interassay variations 2.3% and 5.0%, respectively).

### Assessment of renal histological injury.

Of the 50 subjects with CKD included in the study, 14 had a diagnosis of systemic vasculitis and had had a renal biopsy in the preceding 12 months; all of these were considered adequate for histological assessment (46).

### Measurement of arterial stiffness.

PWV was measured by the foot-to-foot wave velocity method using the SphygmoCor system (SphygmoCor Mx, AtCor Medical, version 6.31), in which a high-fidelity micromanometer (SPC-301, Millar Instruments) was used to determine carotid-femoral PWV (16).

### Data storage and statistical analysis.

This study was powered on the basis of a healthy subfoveal choroidal thickness of 235 ± 53 μm (47) and a healthy temporal RNFL thickness of 79 ± 16 μm (19). To detect a 15% difference in choroidal thickness from normal in those with hypertension or CKD with 90% power and at significance level *P* = 0.05, we needed to include ~22 subjects; for RNFL thickness the corresponding figure was ~30 subjects. Thus, we aimed for 35 subjects in each group. Data were stored and analyzed in Prism, version 6.0 (GraphPad Software Inc.). Data are presented as mean ± standard deviation (SD). Thicknesses were examined by 2-way ANOVA, comparing thicknesses between all 3 patient groups at each macular location using Tukey correction for multiple comparisons. For blood measurements, differences between groups were assessed using parametric or nonparametric analyses as appropriate and similarly, correlation coefficients calculated using the Pearson or Spearman method as appropriate. Stepwise linear regression was used for multivariable analysis using variables showing linear relationship with choroidal thickness (age, CRP, urine P:Cr, IL-6, ET-1, and ADMA). Significance was set at a *P* value of less than 0.05. To measure the sensitivity and specificity of macular volume and subfoveal choroidal thickness at different values, a conventional ROC curve was generated using healthy subjects as controls. The area under the curve was calculated to ascertain the quality of these OCT metrics as biomarkers of CKD. An area of 0.5 is no better than expected by chance, whereas a value of 1.0 signifies a perfect biomarker.

### Study approval.

The clinical study was carried out with the approval of the local research ethics committee and the written informed consent of each subject. The investigations conformed to the principles outlined in the Declaration of Helsinki.

## Author contributions

ND, DJW, BD, SC, JRC, SB, and DT designed the study. CB, JJMHvB, TR, JRC, RK, RM, AC, MKH, PJG, IMM, TMCP, and LW performed the study and carried out the blood analyses. CR and FS performed the animal studies and analyzed the data. MT analyzed the data. All authors were involved in the writing and critical appraisal of the manuscript.

## Supplementary Material

Supplemental data

ICMJE disclosure forms

## Figures and Tables

**Table 3:**
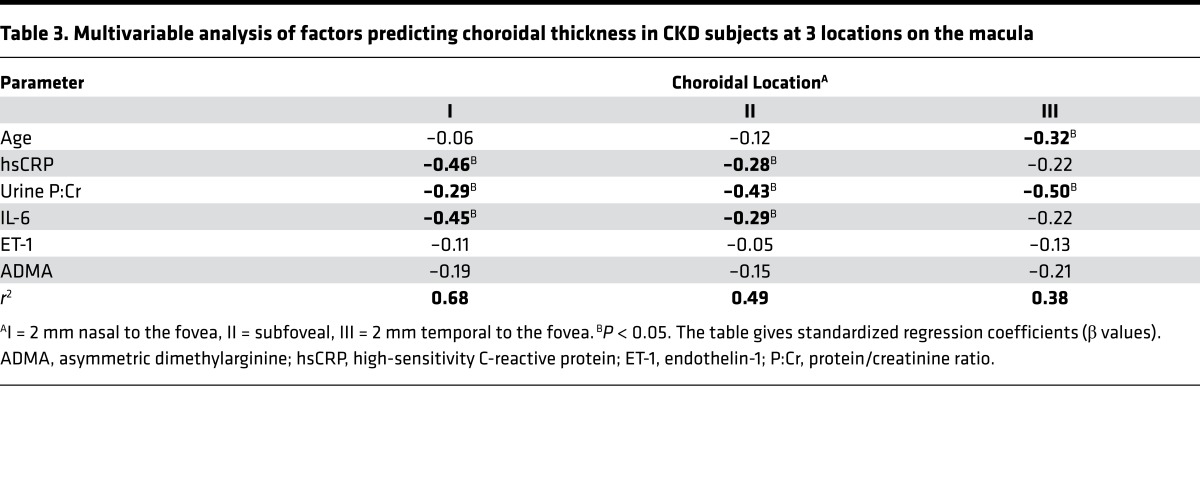
Multivariable analysis of factors predicting choroidal thickness in CKD subjects at 3 locations on the macula

**Table 2:**
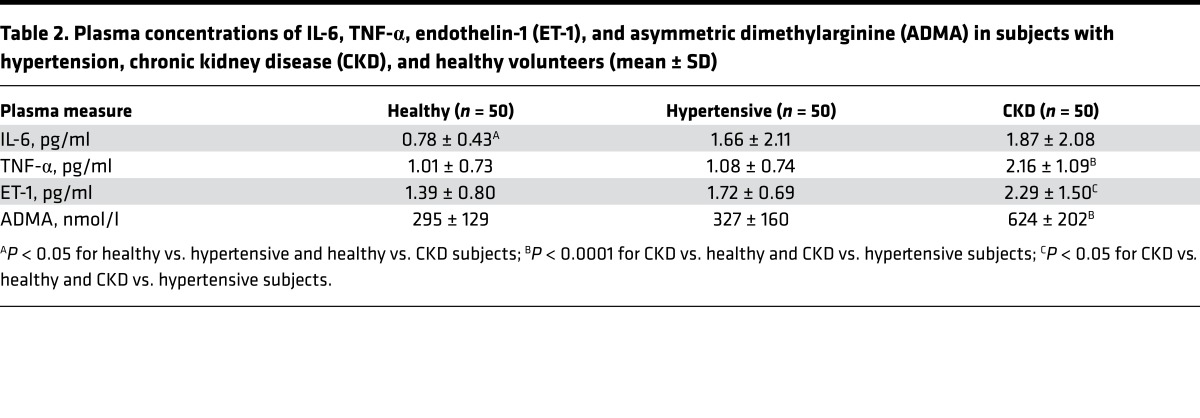
Plasma concentrations of IL-6, TNF-α, endothelin-1 (ET-1), and asymmetric dimethylarginine (ADMA) in subjects with hypertension, chronic kidney disease (CKD), and healthy volunteers (mean ± SD)

**Table 1:**
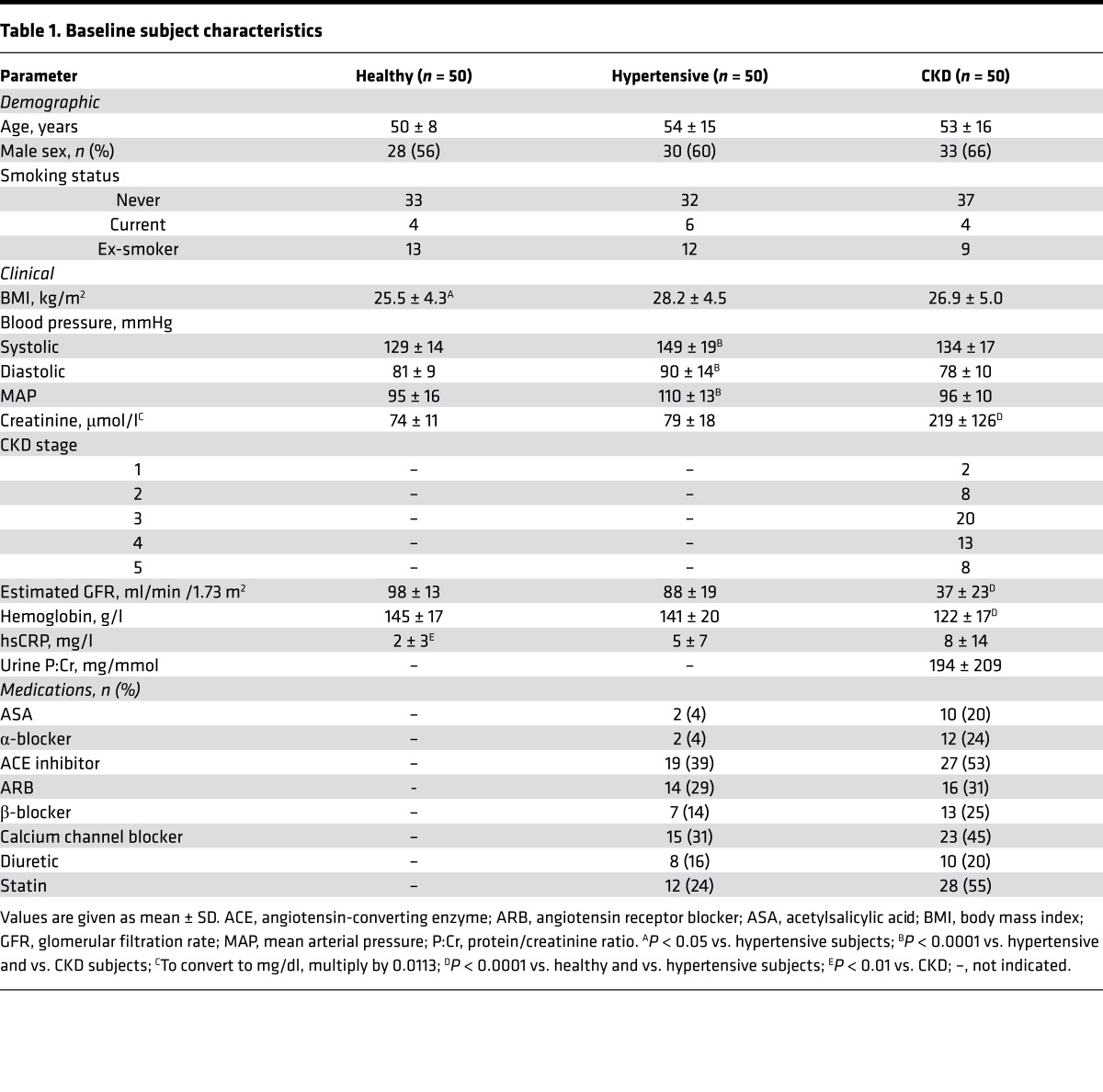
Baseline subject characteristics
