# Supplementary material for: Chorioretinal thinning in chronic kidney disease links to inflammation and endothelial dysfunction
Source: JCI Insight. 2016 Dec 8;1(20):e89173. doi: 10.1172/jci.insight.89173 (PMC5135281; doi:10.1172/jci.insight.89173)
Supplement: Supplemental data [file jciinsight-1-89173-s001.pdf]

Chorioretinal thinning in chronic kidney disease links to systemic inflammation & endothelial dysfunction

Supplemental Information

Craig Balmforth[1*]
Job J M H van Bragt[1*]
Titia Ruijs[1*]
James R Cameron[2]
Robert Kimmitt[1]
Rebecca Moorhouse[1]
Alicja Czopek[1]
May Khei Hu[1]
Peter J Gallacher[1]
Shyamanga Borooah[2, 3]
Iain M MacIntyre[1]
Tom M C Pearson[2]
Laura Willox[4]
Dinesh Talwar[4]
Muriel Tafflet[5]
Christophe Roubeix[6]
Florian Sennlaub[6]
Siddharthan Chandran[2]
Baljean Dhillon[2, 3]
David J Webb[1]
Neeraj Dhaun[1] (*Joint first authors)

[1]BHF Centre of Research Excellence, University of Edinburgh, The Queen's Medical Research Institute, 47 Little France Crescent, Edinburgh, UK.

[2]Anne Rowling Regenerative Neurology Clinic, Centre for Clinical Brain Sciences, University of Edinburgh, Edinburgh, UK.

[3]Princess Alexandra Eye Pavilion, Chalmers Street, Edinburgh, UK.

[4]Department of Clinical Biochemistry & Metabolic Medicine, Royal Infirmary of Glasgow, UK.

[5]INSERM Unit 970, Paris Cardiovascular Research Center – PARCC & Descartes University, Paris, France.
[6]Institut National de la Santé et de la Recherche Médicale, Institut de la Vision, Paris, France.

Supplemental methods

Model of hypertension ± renal injury

The 129/Sv mouse strain is recognized to be more susceptible to injury than the C57BL/6J strain.[1] Using a model of immune-mediated endothelial injury (nephrotoxic nephritis) we have previously shown that 129/Sv mice are more prone to glomerular injury than their C57BL/6J counterparts.[2] For the current study we utilized the angiotensin II (Ang II) infusion model of hypertension in the disease-susceptible 129/Sv and more resistant C57BL/6J mouse strains to generate a model of hypertension alone (BL6 mice) or matched hypertension with associated renal injury (129/Sv mice) (**Supplementary figures 5A & 5B**). Mice were fed a high-salt diet (NaCl 3%) and implanted with subcutaneous osmotic mini pumps (Alzet, model 2002), which infused Ang II (Sigma Aldrich, A9525) continuously (1µg/kg/min) for 1 week. Animals had free access to water. Renal injury was characterised by an increased blood urea nitrogen, proteinuria and glomerulosclerosis.

Mouse OCT

Pupils were dilated with tropicamide (Mydriaticum®, Théa, France) and phenylephrine (Néosynephrine®, Europhta, France). Animals were then anesthetized by isoflurane inhalation (Axience, France) and placed in front of the SD-OCT imaging device (Bioptigen, 840nm HHP; Bioptigen, North Carolina, USA). Eyes were kept moist with 0.9 NaCl throughout the procedure. Acquired images were saved as .avi files and processed with Fiji software. Image artifacts due to respiration were first eliminated using the StackReg Plugin. Then, each movie was converted into a single image by compiling a Z-projection of all the images. Choroidal thickness was measured manually on this maximum projection image in an axis perpendicular to the individual layers at 250 and 600µm from the center of the optic nerve and an average taken of these two readings. OCT was performed in C57BL/6J and 129/Sv mice prior to and following 7 days of Ang II.

Supplementary table 1: CKD subjects' diagnoses. IgA: immunoglobulin-A; ADPKD: autosomal dominant polycystic kidney disease.

**Aetiology for CKD**	**Number of subjects with diagnosis**
Systemic vasculitis	14
IgA nephropathy	8
ADPKD	7
Lupus nephritis	5
Hypertensive nephrosclerosis	5
Membranous glomerulopathy	3
Renal artery stenosis	2
Cystic renal disease	1
Obstructive uropathy	1
Interstitial nephritis	1
Minimal change disease	1
Hyperoxaluria	1
Fabry's disease	1

Supplementary table 2: macular volume and subfoveal choroidal thickness test characteristics for discriminating CKD from health at different cutoff values. CI: confidence interval.

Cutoff for macular volume (mm^3)	Sensitivity	95% CI	Specificity	95% CI
<9.09	1.00	0.93 – 1.00	0.08	0.02 – 0.20
<8.59	0.80	0.66 – 0.90	0.73	0.58 – 0.85
<8.43	0.60	0.45 – 0.74	0.85	0.72 – 0.94
<8.28	0.40	0.26 – 0.85	0.94	0.83 – 0.99
<8.20	0.26	0.15 – 0.40	1.00	0.93 – 1.00
**Cutoff for subfoveal choroidal thickness (µm)**	**Sensitivity**	**95% CI**	**Specificity**	**95% CI**
<437	1.00	0.92 – 1.00	0.13	0.05 – 0.25
<330	0.91	0.80 – 0.98	0.48	0.33 – 0.63
<270	0.70	0.55 – 0.83	0.77	0.63-0.88
<244	0.52	0.36 – 0.66	0.85	0.72 – 0.94
<202	0.26	0.14 – 0.40	0.94	0.83 – 0.99
<105	0.02	0.00 – 0.11	1.00	0.93 – 1.00

Video legends

Video 1: Right eye OCT profiles of macula volume scan (30º x 25º, 61 horizontal sections, 120μm separation) in a healthy subject;

Video 2: Right eye OCT 3D reconstruction of volume scan in a healthy subject

Video 3: Right eye OCT profiles of macula volume scan (30º x 25º, 61 horizontal sections, 120μm separation) in a CKD subject

Video 4: Right eye OCT 3D reconstruction of volume scan in a CKD subject

Figure legends

Supplementary figure 1: Correlation of choroidal thickness (at locations I, II and III on the macula), with estimated glomerular filtration rate (eGFR) in all subjects. Correlation coefficients are Pearson's coefficients.

Supplementary figure 2: Principal component analysis for the factors associated with choroidal thickness in subjects with CKD.

Supplementary figure 3: Renal biopsy assessment. Panel A shows a glomerulus (H&E stain, x200 original magnification) with a focal necrotizing lesion (yellow arrow); panel B shows a glomerulus (H&E stain, x200 original magnification) with a cellular crescent (yellow arrow). 14 biopsies from 14 subjects with ANCA vasculitis were assessed. Correlations of choroidal thickness (at locations I, II and III on the macula) with % of focal necrotizing lesions and cellular crescents (panels C & D, respectively). Correlation coefficients are Spearman's coefficients.

Supplementary figure 4: Correlation of choroidal thickness (at locations I, II and III on the macula), with pulse wave velocity in 20 CKD subjects. Correlation coefficients are Spearman's coefficients.

Supplementary figure 5: Blood urea nitrogen (BUN) (A) and albuminuria (B) in C57BL/6J (blue) and 129/Sv (red) mice following 7 days infusion of Ang II. Cross-sectional image from a mouse OCT (A). The red arrows show the choroidal thickness at 250 and 600µm from the center of the optic nerve. Choroidal thickness in C57BL/6J and 129/Sv mice at baseline (BL) and following 7 days of Ang II infusion (D7); n=4 for each group.

Supplementary figure 1

Choroidal Location I

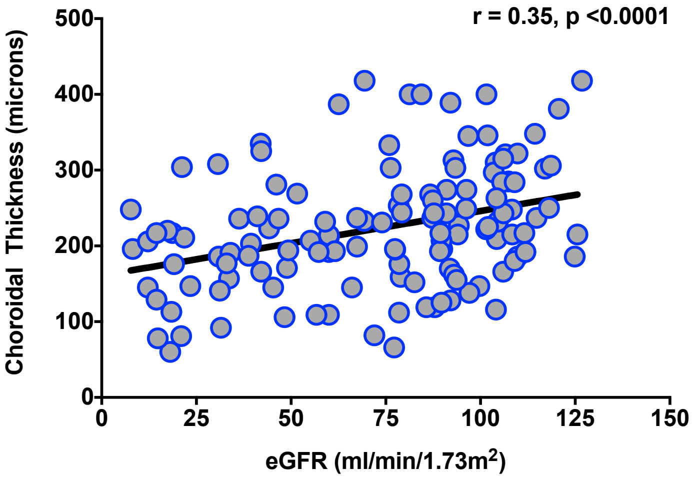

Choroidal Location II

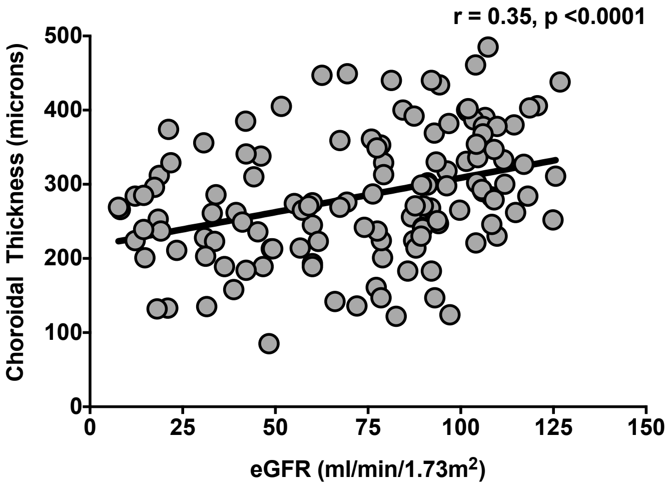

Choroidal Location III

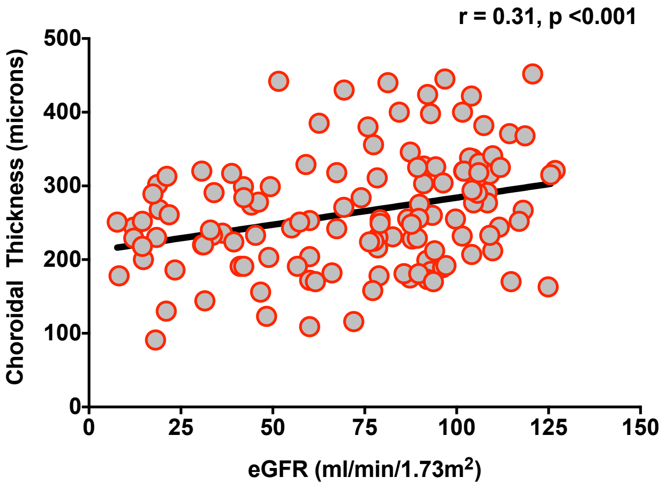

Supplementary figure 2

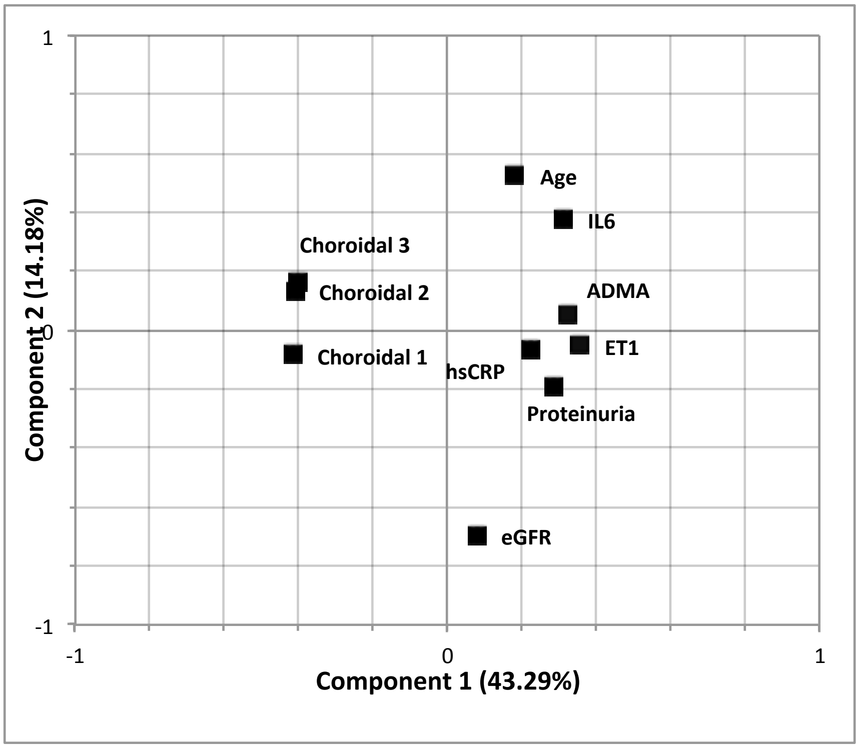

Supplementary figure 3

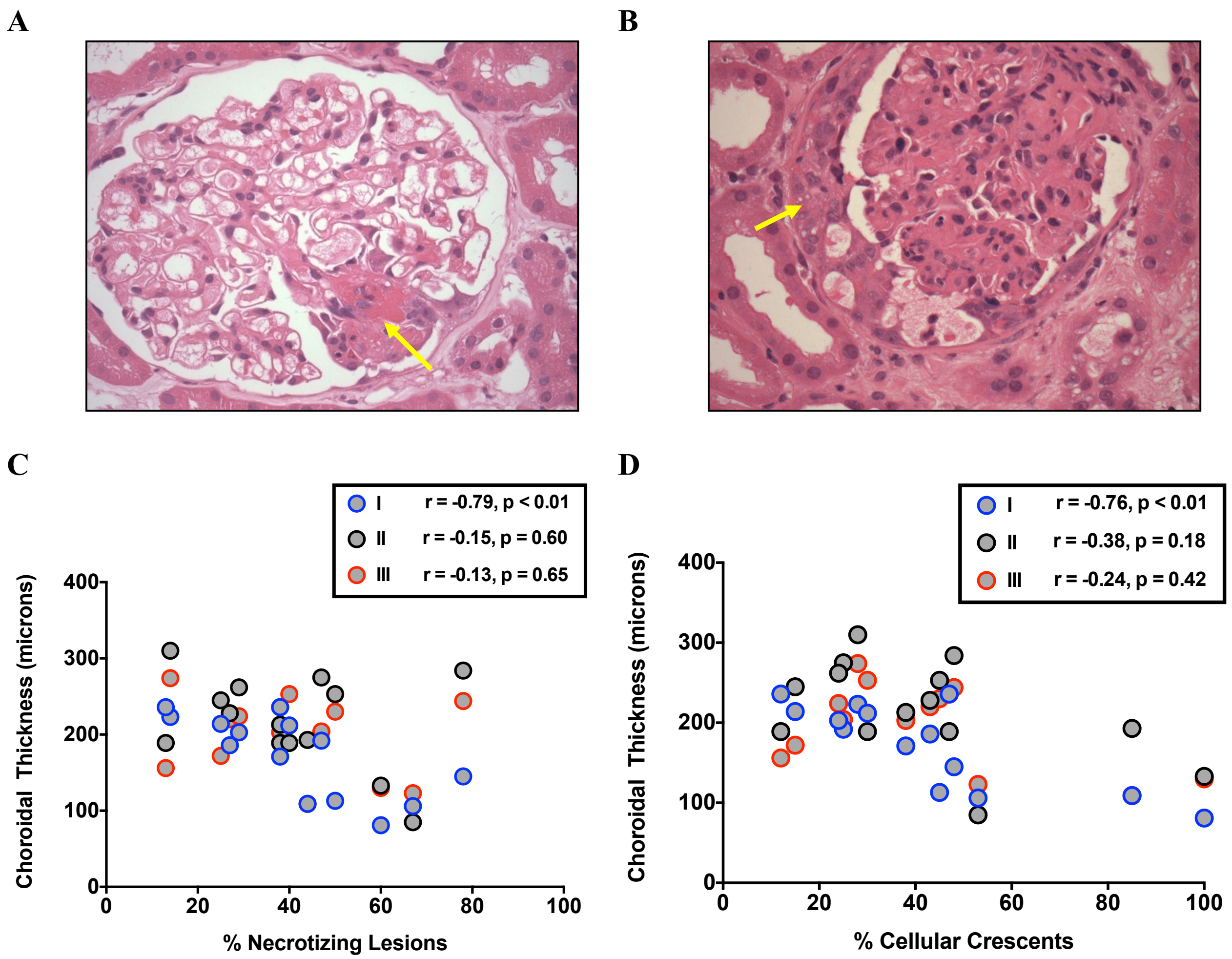

Supplementary figure 4

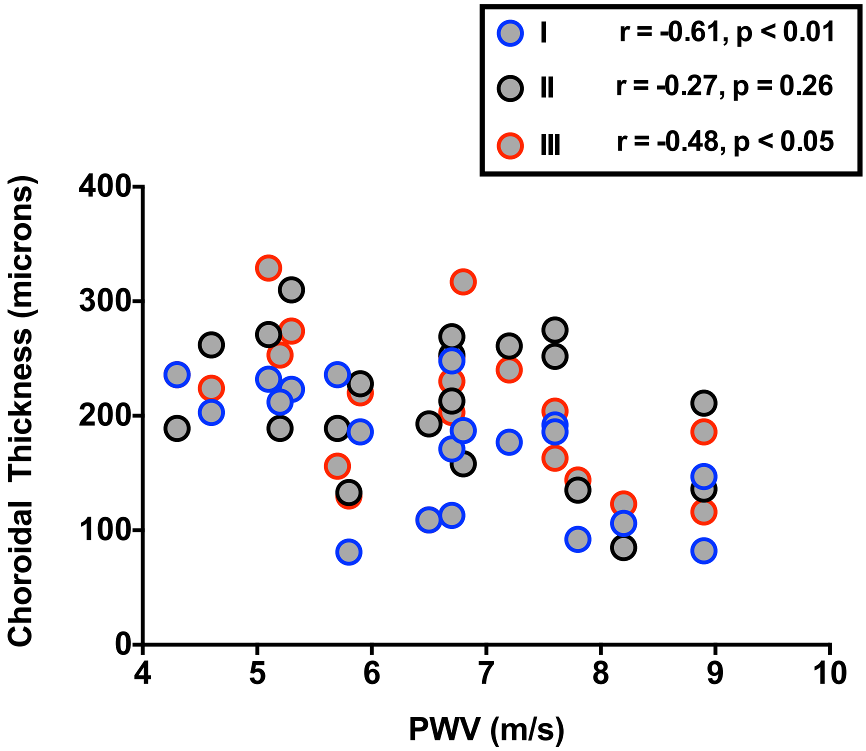

Supplementary figure 5

A

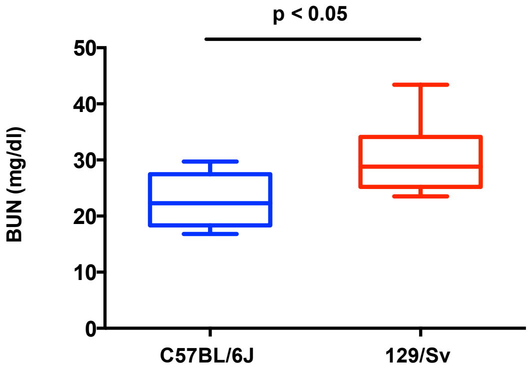

B

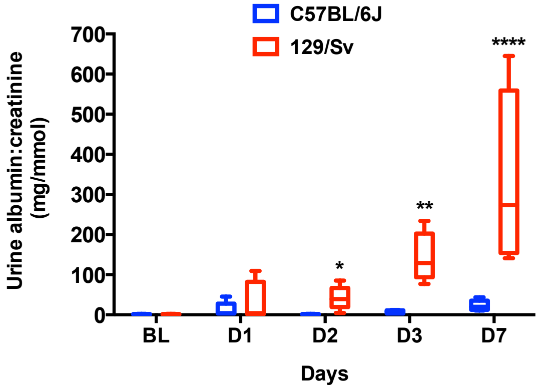

C

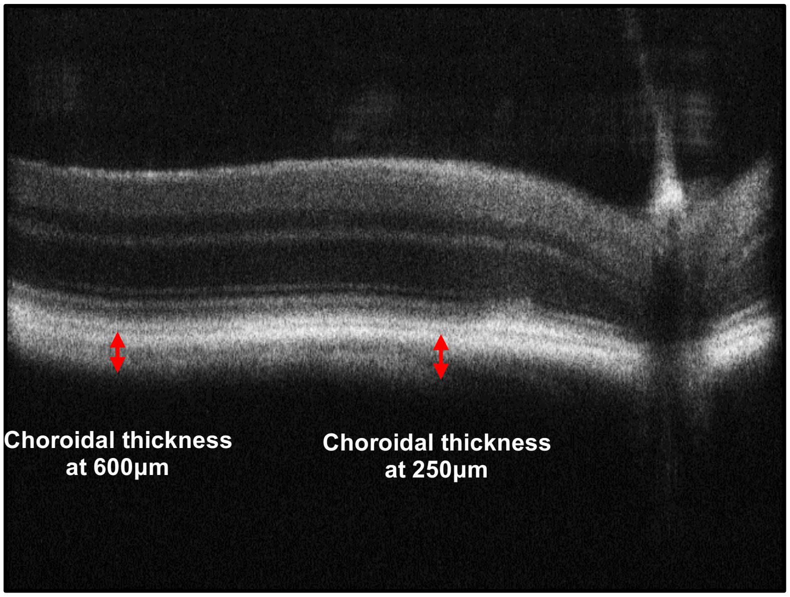

D

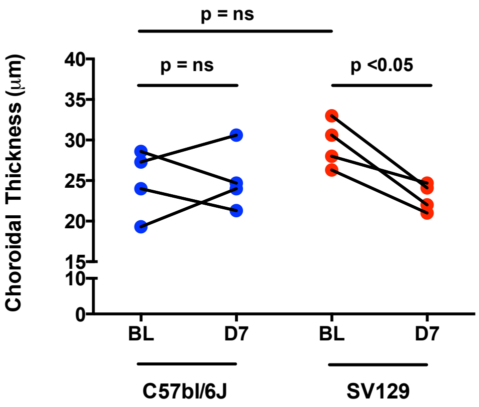

References

1. Xie C, Sharma R, Wang H, Zhou XJ, Mohan C. Strain distribution pattern of susceptibility to immune-mediated nephritis. J Immunol 2004;172:5047-55.

2. Mesnard L, Cathelin D, Vandermeersch S, et al. Genetic background-dependent thrombotic microangiopathy is related to vascular endothelial growth factor receptor 2 signaling during anti-glomerular basement membrane glomerulonephritis in mice. Am J Pathol 2014;184:2438-49.
